# Quantitative Nucleotide Level Analysis of Regulation of Translation in Response to Depolarization of Cultured Neural Cells

**DOI:** 10.3389/fnmol.2017.00009

**Published:** 2017-01-27

**Authors:** Jasbir S. Dalal, Chengran Yang, Darshan Sapkota, Allison M. Lake, David R. O'Brien, Joseph D. Dougherty

**Affiliations:** ^1^Department of Genetics, Washington University School of MedicineSt. Louis, MO, USA; ^2^Department of Psychiatry, Washington University School of MedicineSt. Louis, MO, USA

**Keywords:** activity, depolarization, neuronal, glial, regulation, translation

## Abstract

Studies on regulation of gene expression have contributed substantially to understanding mechanisms for the long-term activity-dependent alterations in neural connectivity that are thought to mediate learning and memory. Most of these studies, however, have focused on the regulation of mRNA transcription. Here, we utilized high-throughput sequencing coupled with ribosome footprinting to globally characterize the regulation of translation in primary mixed neuronal-glial cultures in response to sustained depolarization. We identified substantial and complex regulation of translation, with many transcripts demonstrating changes in ribosomal occupancy independent of transcriptional changes. We also examined sequence-based mechanisms that might regulate changes in translation in response to depolarization. We found that these are partially mediated by features in the mRNA sequence—notably upstream open reading frames and secondary structure in the 5′ untranslated region—both of which predict downregulation in response to depolarization. Translationally regulated transcripts are also more likely to be targets of FMRP and include genes implicated in autism in humans. Our findings support the idea that control of mRNA translation plays an important role in response to neural activity across the genome.

## Introduction

Stimulated neurons show an activity-mediated gene expression program that ultimately results in the remodeling of brain circuitry (Goelet et al., 1986; Sheng and Greenberg, 1990). Because this program contributes to essential functions such as learning and memory, extensive transcriptomic studies *in vitro* and *in vivo* have defined the genes transcribed in response to neuronal activity as well as molecular mechanisms regulating such activity-dependent transcription (Ghosh and Greenberg, 1995; Kim et al., 2010; West and Greenberg, 2011; Malik et al., 2014). In contrast, our knowledge of post-transcriptional regulation, notably activity-dependent changes in translation, is far less comprehensive. Understanding activity-dependent changes in translation is important because new protein synthesis is associated with long-term memory (Flexner et al., 1965; Goelet et al., 1986), synaptic plasticity (Kang and Schuman, 1996; Sutton and Schuman, 2006), growth cone guidance (Campbell and Holt, 2001; Yao et al., 2006), and when dysregulated, neurodegenerative diseases (Wolozin, 2012; Ishimura et al., 2014). Indeed, memory consolidation is blocked when translation is inhibited (Flexner et al., 1965; Nader et al., 2000) but not when transcription is inhibited (Squire and Barondes, 1970), suggesting that post-transcriptional gene regulation alone is sufficient for formation of new memories and that widespread regulation of translation may be pervasive in the central nervous system (CNS). While the basic machinery of translation is common to all cells, it is regulated by myriad factors, including miRNAs and RNA-binding proteins (RBP) (Kozak, 1992; Gebauer and Hentze, 2004; Sonenberg and Hinnebusch, 2009) which may exhibit brain-specific expression (Dittmar et al., 2006; Nowak and Michlewski, 2013; Ishimura et al., 2014), highlighting the importance of studying regulation of translation in CNS derived cells. Recent work has identified some genes that show altered translation in response to experience dependent transient neural activity *in vivo* (Cho et al., 2015). However, the relative magnitude of transcriptional vs. translational regulation for gene expression has not yet been defined.

Translation is subject to regulation at all steps—initiation, elongation and termination—but most stringently at initiation. Initiation involves first scanning of the 5′ untranslated region (UTR) by a pre-initiation complex (PIC). When the PIC encounters the start codon (AUG), it recruits a 60S subunit and begins translation. Several cis-regulatory features present in the mRNA can influence scanning and hence regulate initiation (Sonenberg and Hinnebusch, 2009). These include upstream open reading frames (uORFs) with AUG codons in the 5′UTR that act as sinks for the scanning PIC (Calvo et al., 2009); 5′UTR secondary structures that can hinder the loading of the PIC onto the 5′UTR and subsequent scanning (Babendure et al., 2006); and Kozak consensus sequences which facilitate the recognition of appropriate AUGs by the PIC (Kozak, 2002). Recent work using ribosome profiling/ribosome footprinting (RF) (Ingolia et al., 2009), the deep sequencing of ribosome-protected RNA fragments to quantify ribosomal occupancy across transcripts, has led to new insights in this area. Unanticipated observations from RF in non-CNS systems include widespread use of alternative initiation codons (Ingolia et al., 2009; Lee et al., 2012), and utilization of uORFs in 5′UTRs (Ingolia et al., 2011). However, the impact of uORFs in the 5'UTRs on regulation of translation in response to neural activity has not yet been examined.

Here we studied regulation of translation in mixed neuron-glia cultures, *in vitro* paradigms known to allow robust synaptic maturation of neurons (Eroglu and Barres, 2010). Specifically, we paired RF and RNAseq with KCl depolarization of these cultures, to quantitatively compare the extent of transcriptional vs. translational regulation, and to identify elements that might mediate translational changes specifically. We found that: (i) an even higher proportion of genes are altered translationally than is evident from transcription alone, (ii) overall translation is reduced in response to sustained neuronal stimulation, (iii) as many as 40% of mRNAs showing a change in translation do so independently of changes in mRNA levels, (iv) models taking into account 5′UTR secondary structure and uORFs together can explain a portion of this regulation, and (v) downregulated transcripts are significantly enriched in targets of the RBP *Fragile X Mental Retardation Protein* (FMRP).

## Materials and methods

### Animal research committees

All procedures involving animals were approved by the Animal Studies Committee of Washington University in St. Louis.

### Culture

Primary CNS cells were isolated from cortices of P0 FVB mouse pups of both sexes. Pups were euthanized and cortices were dissected in Hanks's Balanced Salt Solution with glucose and chopped into small pieces with a sterile scalpel blade. The tissue was dissociated in papain for 30 min at 37°C. After inactivating papain, tissue was triturated using a fire polished sterile glass pipette and re-suspended cells were maintained in Neurobasal medium supplemented with B27, 2 mM L-glutamine, 5% heat inactivated horse serum, 100 U/ml penicillin and 100 ug/ml streptomycin. Live cells were counted by staining with trypan blue dye and approximately 7.5 million cells were seeded per well on a 6-well tissue culture plate previously coated with poly-L-ornithine at 0.1 mg/ml (Sigma P4957) and mouse laminin at 10 ug/ml (Invitrogen). Cells were maintained at 37°C, 5% CO_2_, changing half of the medium every second day for 7 days to allow the expansion of glia and maturation of neurons. After 7 days *in vitro*, cells were depolarized by adding KCl to a final concentration of 55 mM in the medium for 3 h. Cells were treated with cycloheximide (100 ug/ml; Sigma) for 7 min before lysis was performed for RNAseq and RF.

### Immunofluorescence

Sterile coverslips coated with poly-L-ornithine and mouse laminin were taken in a 12-well plate, and primary CNS cells were cultured as described above. On day 7, cultures were fixed with 4% paraformaldehyde at room temperature for 20 m and washed twice with phosphate buffer saline (PBS). Cultures were then blocked with 5% normal donkey serum in 0.3% Triton® X-100 at room temperature for 1 h, incubated with primary antibody in block at 4°C overnight, washed three times with PBS, incubated with Alexa fluorophore-conjugated secondary antibodies (1:500, Invitrogen) in block at room temperature for 1 h, washed twice with PBS, incubated with DAPI (Sigma, D9542, 300 nM) at room temperature for 5 m, washed twice with PBS, and finally mounted for confocal imaging. Primary antibodies and dilutions were: Mouse anti-NeuN (Millipore, MAB337, 1:500), Rabbit anti-Aqp4 (SantaCruz, sc-20812, 1:100), and Mouse anti-GFAP (Sigma, G3893, 1:200).

### RF and RNAseq library construction

RF was conducted as described by Ingolia et al. (2011). Briefly, cell lysates were treated with DNase and clarified, and a portion was taken for RNAseq analysis. Remainder was treated with RNase I (100 U/ul; Invitrogen) for 45 min at room temperature, followed by inactivation of the RNase I with SUPERase-In (Thermo Fisher). Ribosomes and protected mRNA fragments were then pelleted with ultracentrifugation on 1 M Sucrose cushion at 200,000 g for 4 h at 4°C. RNA was isolated from the pellet using miRNeasy kit from Qiagen, and ribosome protected fragments (26–34-nucleotides (nt) size) were selected on a 15% polyacrylamide TBE-urea gel. After dephosphorylation, linker ligation was performed at room temperature for 2.5 h using miRNA cloning linker (NEB). Linker ligated product was separated from unligated product on a 15% polyacrylamide gel, and reverse transcription was performed using SuperScript III (Invitrogen). Leftover RNA was hydrolyzed with 1N NaOH and the cDNA was circularized using CircLigase (Epicentre). Circularized cDNA was subjected to rRNA depletion using subtractive hybridization with biotinylated rRNA oligo pool. The depleted product was PCR amplified using Phusion polymerase (NEB) and different indexing primers and the final product was purified on 8% polyacrylamide non-denaturing gels. Libraries were analyzed for concentration and fragment size using a high-sensitivity DNA chip on the Agilent BioAnalyzer, and then pooled and sequenced on two lanes of an Illumina Hiseq 2000 system (50 bp, single end).

For RNASeq libraries, DNAse I treated total RNA was purified with RNeasy MinElute columns (Qiagen). We generated double stranded cDNA using Nugen Ovation RNAseq system V2, starting from 68 ng of total RNA. Standard Illumina sequencing libraries were generated from 1 to 2 ug of cDNA, sheared to ~200 nt, and sequenced (50 bp, single end).

### Analysis of sequence data

Using FastQC (version 0.11.2; Babraham Bioinformatics, 2010 website, http://www.bioinformatics.babraham.ac.uk/projects/fastqc/) we checked the library size of each of the three replicate samples, and found that replicate 3 in the KCl treatment group had a much lower size compared to other two replicates. We removed this sample for the downstream analysis. For remaining libraries, we trimmed the adapters, including PCR primers, using Trimmomatic (version 0.32) (Bolger et al., 2014). Minimum read lengths were set to 25 nt for RF and 20 for RNAseq after trimming adapters, and for RF, the maximum read length was set to 35. For both RF and RNAseq, we removed reads aligning to rRNA sequence as detected by STAR (version 2.3.1z8) (Dobin et al., 2013). Before mapping the reads, we first removed degenerate sequences from the mouse mm10 transcriptome (Downloaded from Ensembl-release 75) as described (Ingolia et al., 2009; Dunn et al., 2013). We then aligned both RF and RNAseq reads to this non-degenerate transcriptome using bowtie2 (version 2.2.2) (Langmead and Salzberg, 2012) retaining both uniquely mapped and multi-mapped reads. Then, we counted both RF and RNAseq reads using the BEDTools (version 2.20.1) (Quinlan and Hall, 2010) intersect command. For calculating translation efficiency (TE), the ratio of the coding sequence RF reads to RNAseq reads, we removed several positions in the transcriptome: 9 nt before the first nt of each start codon, 15 nt after the last nt of each start codon, 15 nt before the first nt of each stop codon, and 15 nt after the last nt of each stop codon, as described (Dunn et al., 2013).

Using RNAseq, we defined the levels of each transcript as the number of reads mapping to the exonic sequence, in counts per million reads (CPM), then normalized for the length of each transcript, in kilobases (RPKM). For RF, we measured ribosome footprint density for coding sequence (CDS) of each transcript, normalized as above. For all downstream analyses, we focused on 6960 transcripts with robust expression, defined by RNAseq and RF RPKM values of ≥ 10 for at least two samples. Consistent with prior studies, the length of most of our protected fragments is around 30 nucleotides—the expected footprint of a ribosome. Differentially transcribed or translated genes were identified using a permuted *t*-test with replacement after integration into a digital gene expression object using the edgeR package (Robinson et al., 2010).

Correlation between mRNA abundance and CDS RF levels was based on the Gamma distribution assumption, and the fit for this generalized linear model was calculated using the definition $R^{2}=1-{SS}_{error}/{SS}_{total}$. For all other linear modeling and ANOVA tests, we ensured that model residuals were approximately normally distributed.

Likely glial and neuronal mRNAs were defined from using the (Zhang et al., 2014) dataset (barreslab_rnaseq.xlsx) from http://web.stanford.edu/group/barres_lab/brain_rnaseq.html. To identify transcripts significantly enriched in each cell type, we used the specificity index (SI) algorithm (Dougherty et al., 2010) with default settings, and a cutoff of *pSI* < 0.05. Raw and analyzed data are available at GEO: GSE77076.

### Metagene analysis

For CDS, in addition to filtering out low expression genes, we also excluded transcripts with a length <2000 nt first. Metagene plots were generated using metagene R package (Beauparlant et al., 2014).

### Linear modeling to predict CDS TE change

Across the 642 transcripts regulated by KCl stimulation, we examined the relationship between log_2_ fold-change in CDS TE and a number of primary sequence features known to regulate translation using linear regression: GC content, number of uAUGs, number of upstream Kozak matches, and length and secondary structure of 5′UTR, CDS, and 3′UTR. To generate a measure of the level of secondary structure, we used the Vienna RNA package (Lorenz et al., 2011) which outputs the free-energy value of the most stable secondary structure for each input sequence. A lower free-energy value indicates a more stable structure.

Specifically, we examined the effect of each predictor in a linear model individually and then used stepwise regression to find the subset of predictors which, when combined in a multivariate linear model, explain the greatest amount of variance in CDS TE change. When choosing the inputs for stepwise regression, we eliminated highly correlated or redundant predictors. For example, we selected number of upstream Kozak matches and omitted number of uAUGs, since the two variables are largely redundant, and the former is a better individual predictor. Additionally, since secondary structure and length were highly correlated (Pearson *r* = 0.94 for 5′UTR, 0.99 for CDS, 0.98 for 3′UTR), we considered a new variable: secondary structure normalized by length. For the 5′UTR, this variable performed better than length or secondary structure alone as an individual predictor and thus was selected as an input to stepwise regression. For the CDS, we selected secondary structure, as it performed better than length or normalized secondary structure in a univariate model. For the 3′UTR, we selected the categorical length variable, as it performed better than continuous length, secondary structure, or normalized secondary structure in a univariate model. Finally, we included a term for the interaction between normalized 5′UTR secondary structure and number of upstream Kozak matches in the stepwise regression input.

### Comparison to miRNA and RBP targets

Predictions for targets of all available mouse miRNAs were downloaded from miRDB (v5.0) (Wong and Wang, 2015). This included 634,009 target predictions for 1912 mouse miRNAs across 18,639 RefSeq transcripts. However, to prevent statistical inflation (Kopp et al., 2015) for all overlap analyses, only the 6960 robustly measurable transcripts were considered. Any target prediction for one of our 6960 transcripts with a confidence of 80 or above was included. BiomaRt was used to map RefSeq IDs from mirDB from Ensembl gene IDs used in our analysis. The set of each miRNAs predicted targets was then tested for overlap with TE regulated genes using a one-tailed Fisher's exact test. Results were corrected for multiple testing correction using Benjamini-Hochberg.

For overlap with CELF4 targets, we used the high-confidence candidates defined by Wagnon et al. (2012), specifically the 2000 highest ranked transcripts from their file S2. For FMRP, we used the 842 transcripts defined as high confidence (*p* < 0.01) targets by Darnell et al. (2011) from their supplemental table 2. For rare *de novo* variant genes in autism, we used the genes in table 4 from Sanders et al. (2015). In each case these were filtered to consider only those transcripts that were robustly measurable in our cultures, then tested using a one-tailed Fisher's exact test for overlap with the translationally regulated genes.

## Results

### Ribosome footprinting of primary cultures

**Figure 1:**
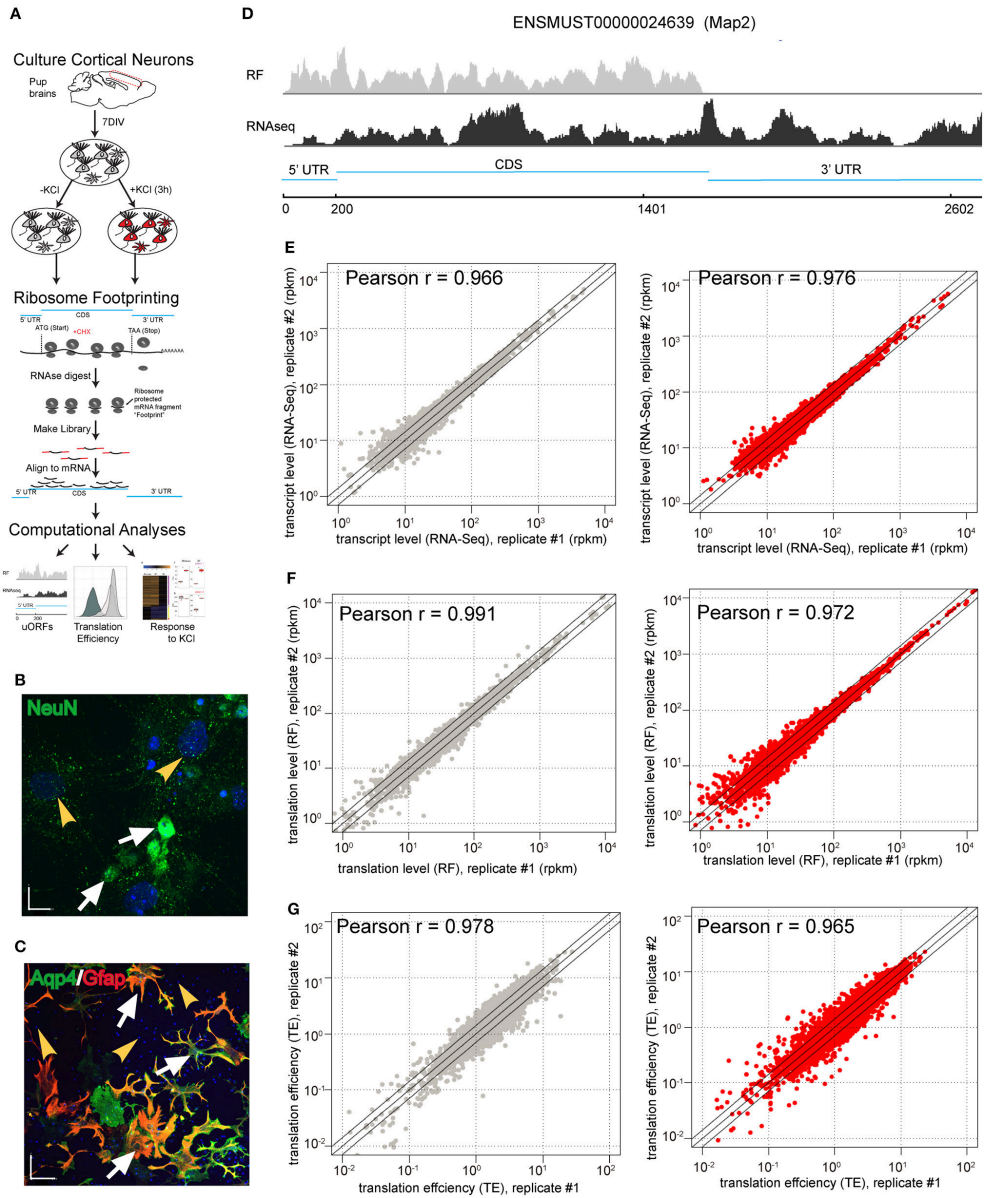
**Ribosome footprinting of primary cultures is reproducible. (A)** Illustration of the experimental design. Primary cultures were derived from mouse brains, matured for 7 days, then replicate cultures were exposed to depolarizing stimuli. Parallel RNAseq (not shown) and RF were conducted to measure transcript abundance and ribosomal occupancy of mRNA, respectively. RF entails harvesting cycloheximide (CHX)-stalled ribosomes, then digesting with RNAse I all mRNA except fragments physically protected by ribosomes. Libraries were prepared from protected fragments and aligned to the transcriptome, enabling downstream computational analyses. **(B,C)** 7-day primary cultures containing a mixture of neurons and glia. **(B)** Immunostaining for NeuN confirms the presence of neurons (white arrows) as well as non-neuronal cells (yellow arrowheads). (Blue is DAPI staining for nuclei; Scale bar = 17 μM) **(C)** Double immunostaining for Aqp4 and GFAP reveals astrocytes (white arrows) and putative non-glial cells (yellow arrowheads). (Blue is DAPI staining for nuclei; Scale bar = 70 μM). **(D)** Screenshot of RNASeq read depth in counts per million (CPM), showing fragments covering the entirety of a representative mRNA (bottom), and RF read depth, showing coverage only of CDS and 5'UTR (top) using *Map2* gene. **(E–G)** Scatterplots comparing the replicate cultures show high levels of reproducibility for **(E)** transcript levels, **(F)** RF densities as well as **(G)** TE in both untreated (gray) and KCl-treated samples (red) (all Pearson *r* > 0.96).

To study regulation of translation in response to neuronal activity, we performed parallel RF and RNAseq in primary neuron-glia mixed cultures (Figures 1A–C). Activity of neurons *in vitro* or *in vivo* has long been known to regulate transcription of specific genes. For example, depolarization of neuronal cultures with KCl induces transcription of immediate early genes such as *c-Fos* (Sheng and Greenberg, 1990; Ghosh and Greenberg, 1995; West et al., 2002). Here, we used the same stimulation paradigm coupled to RF to allow a comparative investigation of translational regulation (Figures 1A,D).

First, we established that RF allows for reproducible measures of translation (ribosomal occupancy) in this culture system. Normalization and removal of transcripts with low read counts as described in prior RF studies (Ingolia et al., 2009, 2011; Dunn et al., 2013; Gonzalez et al., 2014) resulted in 6960 measurable transcripts. Replicates showed high reproducibility in our measures of transcript abundance (RNAseq) or ribosomal occupancy (RF) with or without KCl treatment (Pearson *r* > 0.966, Figures 1E,F). To identify transcripts subject to translation regulation, we also calculated a measure of ribosomal density—“translation efficiency” (TE): the ratio of CDS ribosomal occupancy to transcript abundance. As such, it is an estimate of the number of ribosomes bound to each copy of a particular mRNA in the cells. TE was also highly reproducible (Pearson *r* > 0.965, Figure 1G).

**Figure 2:**
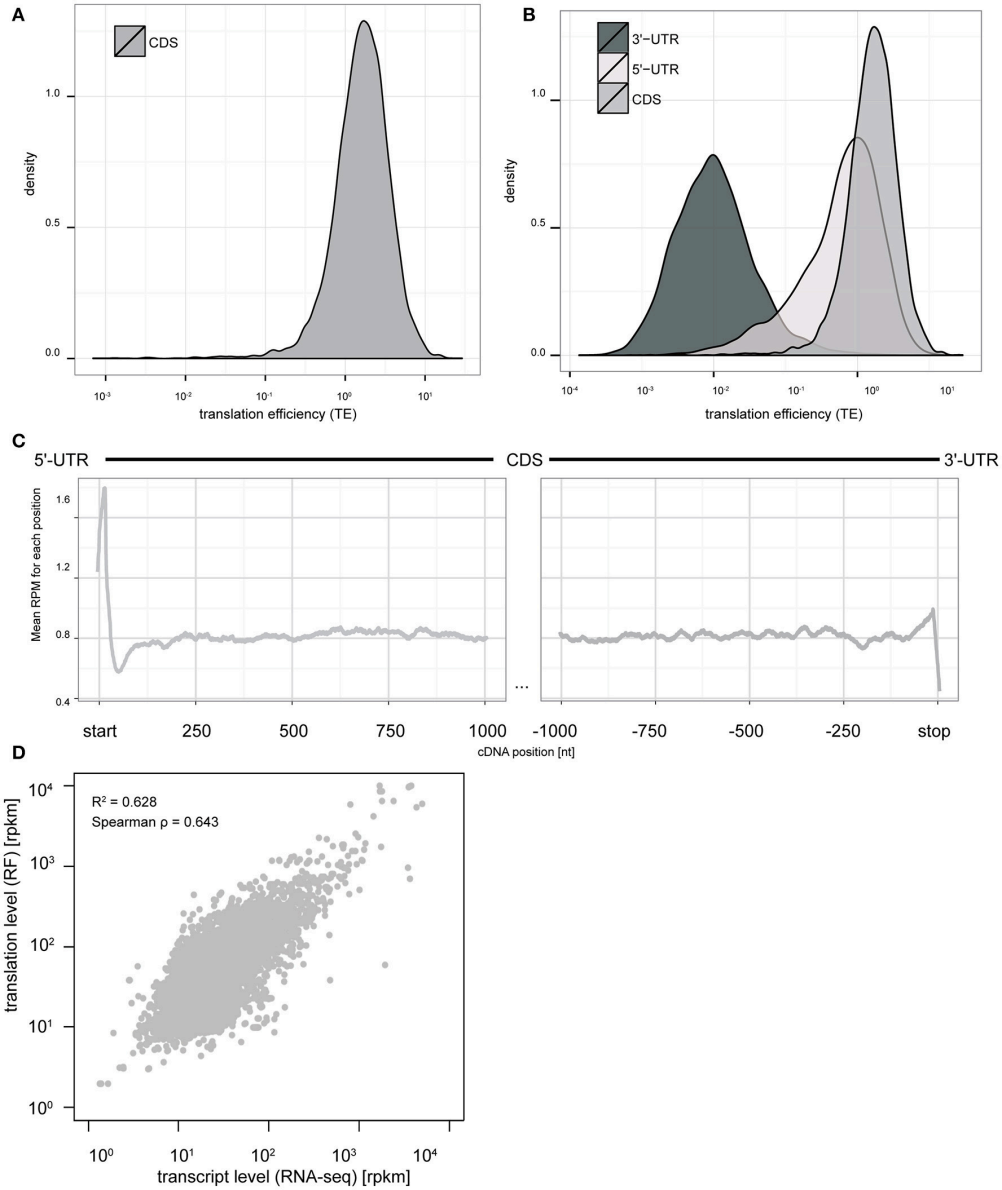
**Ribosome footprinting of primary cultures allows nucleotide resolution analysis of translation levels and efficiency. (A)** The distribution of TE measures across CDS of all transcripts spans more than three orders of magnitude (log_10_ scale). **(B)** Comparison of TE distributions between UTRs and CDS indicates efficient ribosomal occupancy of both CDS and 5′UTR, but not 3′UTR. **(C)** Metagene analysis at nucleotide resolution indicates a peak of ribosome footprints at the start codon and stop codon consistent with initiation and termination being slower than elongation rates (y-axis: mean CPM across all transcripts with CDS length >= 2000 bps. x-axis: nucleotide position relative to start/stop codons). **(D)** Scatterplot comparing transcript levels for mRNAs (RNAseq, in log_10_ RPKM scale) to translation levels (RF, in log_10_ RPKM scale). Fitting a model indicates variation in transcript levels can account for 62.8% of the variance in translation levels (*p* < 2e–16).

Next, we examined the basic features of translation in this culture system. Similar to RF studies in other systems (Ingolia et al., 2009, 2011; Dunn et al., 2013; Gonzalez et al., 2014) the distribution of TE spans more than three orders of magnitude (Figure 2A), indicating substantial differences in baseline TE across different transcripts. We also calculated ribosomal occupancies and densities in the 5′ and 3′UTRs. We found that TEs of the CDS and 5′UTRs are higher than 3′UTR (Figure 2B), consistent with prior studies. Also, a metagene analysis of our RF data showed the expected peaks at both the start codon and stop codons (Figure 2C).

Finally, we sought to compare the relative contribution of transcript abundance vs. translational regulation in the baseline translation levels as estimated from ribosome occupancy. While we found that transcript abundance and ribosomal occupancy are highly correlated (Spearman ρ = 0.643); a generalized linear regression analysis indicates that transcript abundance only predicts ~60% of the overall ribosomal occupancy across genes (*p* < 2e-16, Figure 2D). This indicates that roughly 40% of the variance in translation levels is regulated by mechanisms beyond simple alteration of mRNA abundance.

### KCl stimulation of primary neuron-glia cultures alters translation of specific coding sequences

Next, to quantify the extent of regulation of translation by depolarization, we compared the transcript abundance and ribosomal occupancy of stimulated and unstimulated cultures. This allowed us to examine both the global response to stimulation across the genome and study the responses of individual transcripts.

**Figure 3:**
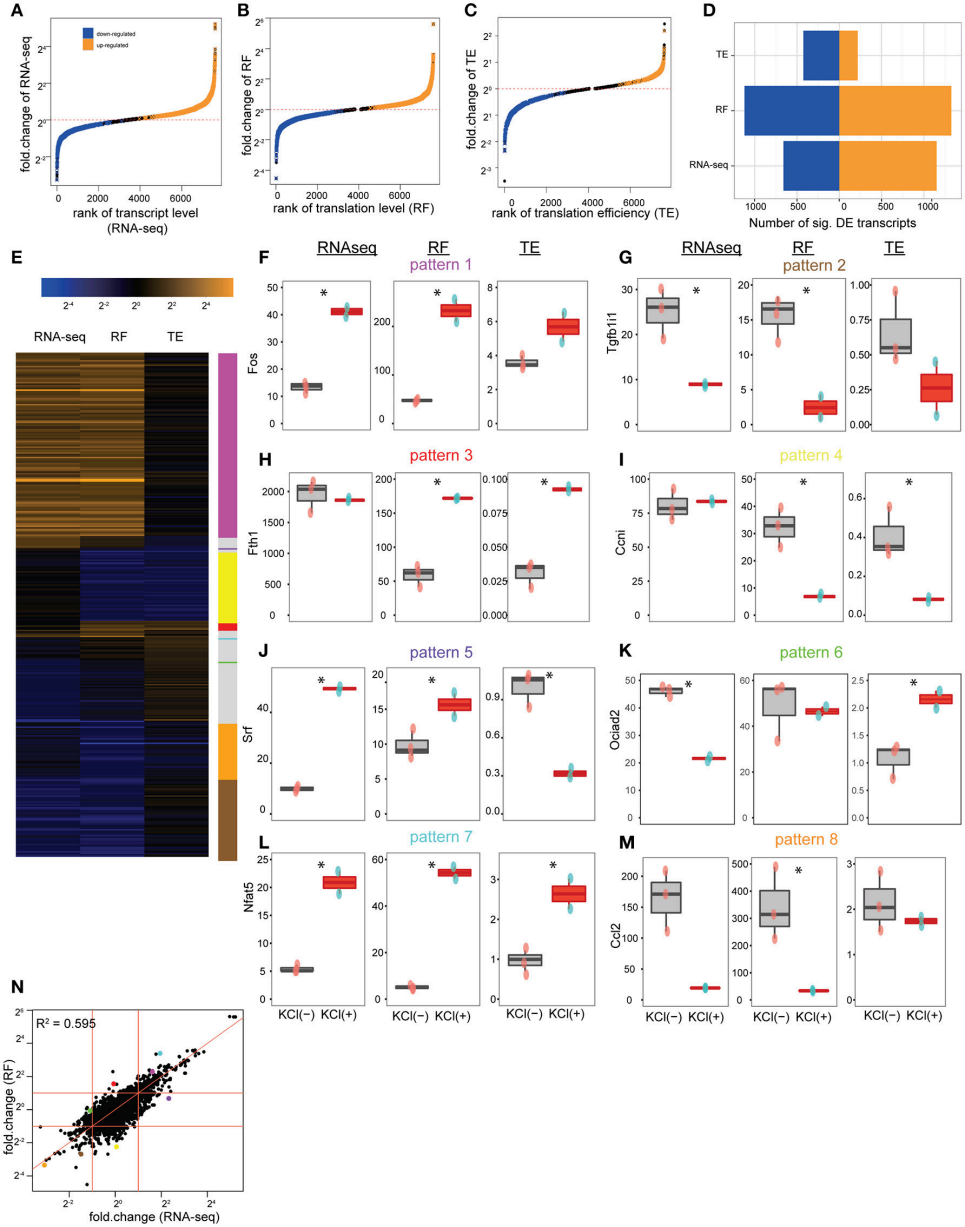
**Depolarization alters the translation of specific transcripts. (A)** Cumulative distribution showing the alteration of transcript levels (RNAseq) by KCl stimulation in culture. One thousand one hundred and fifty-one transcripts, corresponding to 724 genes, were significantly upregulated (orange), and 660 transcripts, corresponding to 451 genes, downregulated (blue) (all unadjusted *p* < 0.05, permuted t-statistic). **(B)** Cumulative distribution showing alteration of translation levels (RF) by KCl stimulation. One thousand three hundred and twenty five transcripts, corresponding to 826 genes, were upregulated, and 1121 transcripts, corresponding to 700 genes, downregulated (all unadjusted *p* < 0.05, permuted t-statistic). **(C)** Cumulative distribution showing alteration of TE by KCl stimulation. Two hundred and eighteen transcripts, corresponding to 164 genes, were upregulated, 424 transcripts, corresponding to 286 genes, downregulated (all unadjusted *p* < 0.05, permuted *t*-statistic). **(D)** Bar plot summarizing number of transcripts being up/downregulated. **(E)** Heatmap of fold-changes (log_2_ scale) for highly altered transcripts (showing those genes with fold-change in top or bottom 5% for RNAseq, RF or TE. In total, there are 344 transcripts as rows, corresponding to 229 unique genes). **(F–J)** KCl stimulation (red boxplots) can separately regulate transcript levels and translation levels of individual CDS shown in E [^*^represents unadjusted *p* < 0.05, permuted t-statistic; num_of_sample = 3 for untreated (pink-dots) and num_of_sample = 2 for KCl-treated (blue-dots) samples]. **(F)** The immediate early gene, *Fos*, is representative of a class of genes (magenta line) where both transcript levels (RNAseq) and translation levels (RF) were increased by KCl stimulation, but TE wasn't significantly affected. **(G)** *Tgfb1i1* exemplifies those CDS (tan line) that decreased transcription and translation levels after KCl stimulation, but didn't alter TE. **(H)** *Fth1* is representative of the class of genes (red line) that specifically increased TE in response to KCl stimulation. **(I)** *Ccni* downregulated translation levels (RF) while transcript levels were constant (yellow line). **(J)** *Srf* (purple line) and **(K)** *Ociad2* (green line) show a homestatic-like response where large magnitude changes in transcript abundance are countered by changes in TE resulting in relatively less change in overall translation (RF) **(L)** *Nfat5* (cyan line) and **(M)** *Ccl2* (orange line) show synergistic-like responses where changes in both transcript abundance and TE occur in concert resulting in an amplification of the change in translation overall. **(N)** Scatterplot comparing the log_2_ fold-changes in transcript levels and translation levels following KCl stimulation. Fitting a linear model indicates that changes in transcript level can account for 59.5% of the change in translation level (*p* < 2e-16). Red diagonal line represents intercept = 0, slope = 1. Red horizontal and vertical lines represent fold-change = 2 and 1/2. Dots represent transcripts highlighted in **(F–M)** with consistent pattern-coding colors.

Overall, KCl stimulation resulted in a significant alteration in the abundance of 1811 transcripts, corresponding to 1175 genes (Figures 3A,D), and the ribosomal occupancy on CDS of 2446 transcripts, corresponding to 1526 genes (Figures 3B,D). Thus, the cultures show more alterations at the level of translation than are apparent at the level of transcription alone. This difference is largely accounted for by the 642 transcripts, corresponding to 450 genes, that significantly alter their TE (Figures 3C,D), indicating substantial activity-dependent regulation of translation of specific transcripts.

Individual transcripts responded to depolarization in a variety of ways, as illustrated in Figure 3E. For example, the immediate early gene *Fos* is induced transcriptionally by depolarization. It is representative of genes (magenta line) where both transcript abundance and ribosomal occupancy were increased in a fairly proportional manner, but TE was not significantly affected (Figure 3F). Thus these genes appear to be upregulated primarily by increasing transcript levels, with increases in translation largely following passively. Likewise, Transforming Growth Factor Beta 1 Induced Transcript 1 (*Tgfb1i1*) is representative of genes (tan line) that are transcriptionally downregulated without significant change in TE, and thus exhibit a simple corresponding downregulation of translation (Figure 3G). In contrast, there are genes that appear to be regulated only via alteration in translation without change in transcript levels (red and yellow lines). For example, administration of iron is known to upregulate Ferritin protein by inducing the recruitment of existing *Fth1* mRNA to ribosomes, thus increasing translation (Zähringer et al., 1976). In our cultures, we see a robust increase in TE of the *Fth1* mRNA in response to stimulation, without change of transcript abundance (Figure 3H), suggesting that one of the effects of KCl stimulation of neurons and glia is enhanced recruitment of *Fth1* mRNA to ribosomes. Similarly, the orphan cyclin *Ccni* (Figure 3I) is representative of translationally downregulated genes with decreased ribosomal occupancy, yet no observed change in transcript abundance measured by RNASeq. Furthermore, there are classes of genes that show even more complicated regulation (purple and green lines). For example, genes such as *Srf* (Figure 3J) or *Ociad2* (Figure 3K) show homeostatic-like regulation, whereby an increase (or decrease) in ribosomal occupancy upon stimulation appears to compensate for a decrease (or increase) in transcription as measured by changes in RNASeq. Thus, RNA abundance can change but putative protein synthesis remains relatively unaltered with no net change of RF levels. Finally, genes like *Nfat5* (Figure 3L) and *Ccl2* (Figure 3M), show an amplification response, whereby both transcript abundance and TE change in concert to either dramatically amplify (cyan line) or suppress (orange line) translation levels.

**Figure 4:**
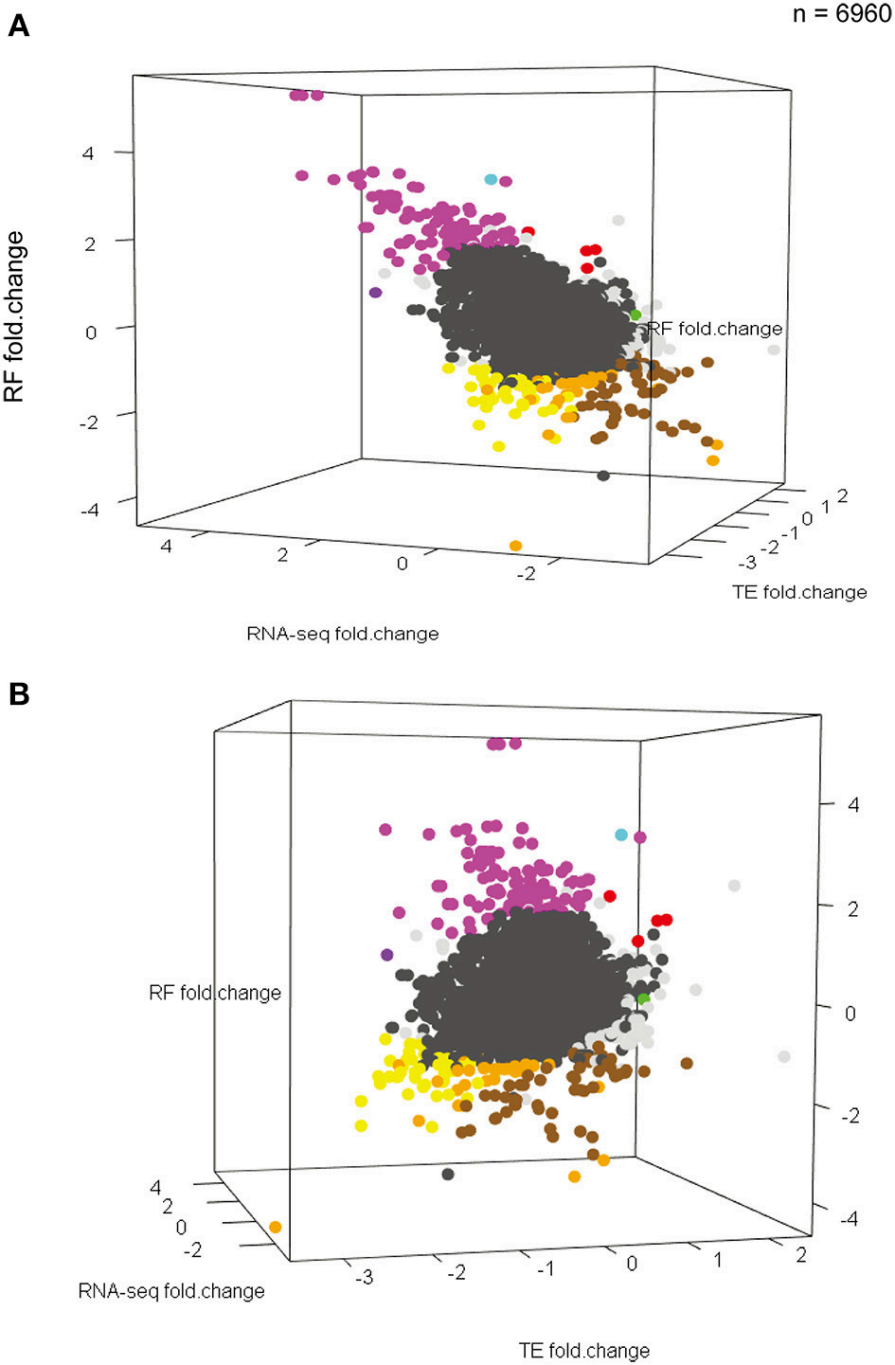
**KCl stimulation results in a continuum of different transcriptional and translational responses. (A,B)** Two different perspectives of a three-dimensional scatterplot showing the distribution of RNAseq changes, RF changes, and TE changes (all in log_2_ scale) in response to KCl stimulation for all 6960 measurable transcripts. Points for transcripts changing significantly in any dimension are bold. Colors correspond to patterns in Figure 3 (Note: Darker-gray dots represent the rest of 6616 transcripts). Though a range of patterns is seen, responses do not fall into apparently discrete clusters.

Overall, this provides additional evidence for the importance of activity-dependent regulation of translation as a mechanism for regulating protein levels following stimulation. Fitting a linear model to predict the fold-change in translation level (RF) by change in transcript abundance indicates that roughly 40% of the change in ribosomal occupancy in response to stimulation is not due to changes in mRNA abundance (Figure 3N). This supports the existence of substantial regulation of translation of specific transcripts in response to activity that parallels the well-characterized epigenetic and transcriptional responses (Sheng and Greenberg, 1990; West et al., 2002; Malik et al., 2014). While we have highlighted examples that have relatively simple interpretations (Figures 3F–M), we note that the responses across transcripts do not fall into discrete clusters, but represent a continuum of different transcriptional and translational responses (Figures 4A,B). Nevertheless, the diversity of different responses indicates that there are multiple mechanisms of translational control downstream of stimulation, and that they are sequence specific.

As our cultures contained both neurons and glia (Figures 1B,C) we also examined separately the responses of transcripts that are likely derived from each cell type. First, using benchmark data from the major CNS cell types *in vivo* (Zhang et al., 2014), we defined sets of transcripts typically enriched in each cell type (e.g., Gfap, in astrocytes, Snap25 in neurons, Table S1), and separately assessed the ability of mRNA abundance to predict ribosome occupancy for the neuronal and glial transcript lists. For the glial specific transcripts, mRNA abundance predicted 67% of the variance in occupancy at baseline and 69% of the change in RF in response to stimulation (Figure S1). Interestingly, for the neuronal specific transcripts, mRNA abundance was less predictive of ribosome occupancy: predicting 43% at baseline and 53% of the change in occupancy with depolarization (Figure S1). This is consistent with post-transcriptional regulation being more important in neurons than in glia generally, with >50% of the variance in the baseline ribosome occupancy on the neuronal transcripts not being explained by transcript abundance.

### UTR sequence mediates changes in CDS translation in response to KCl stimulation

**Figure 5:**
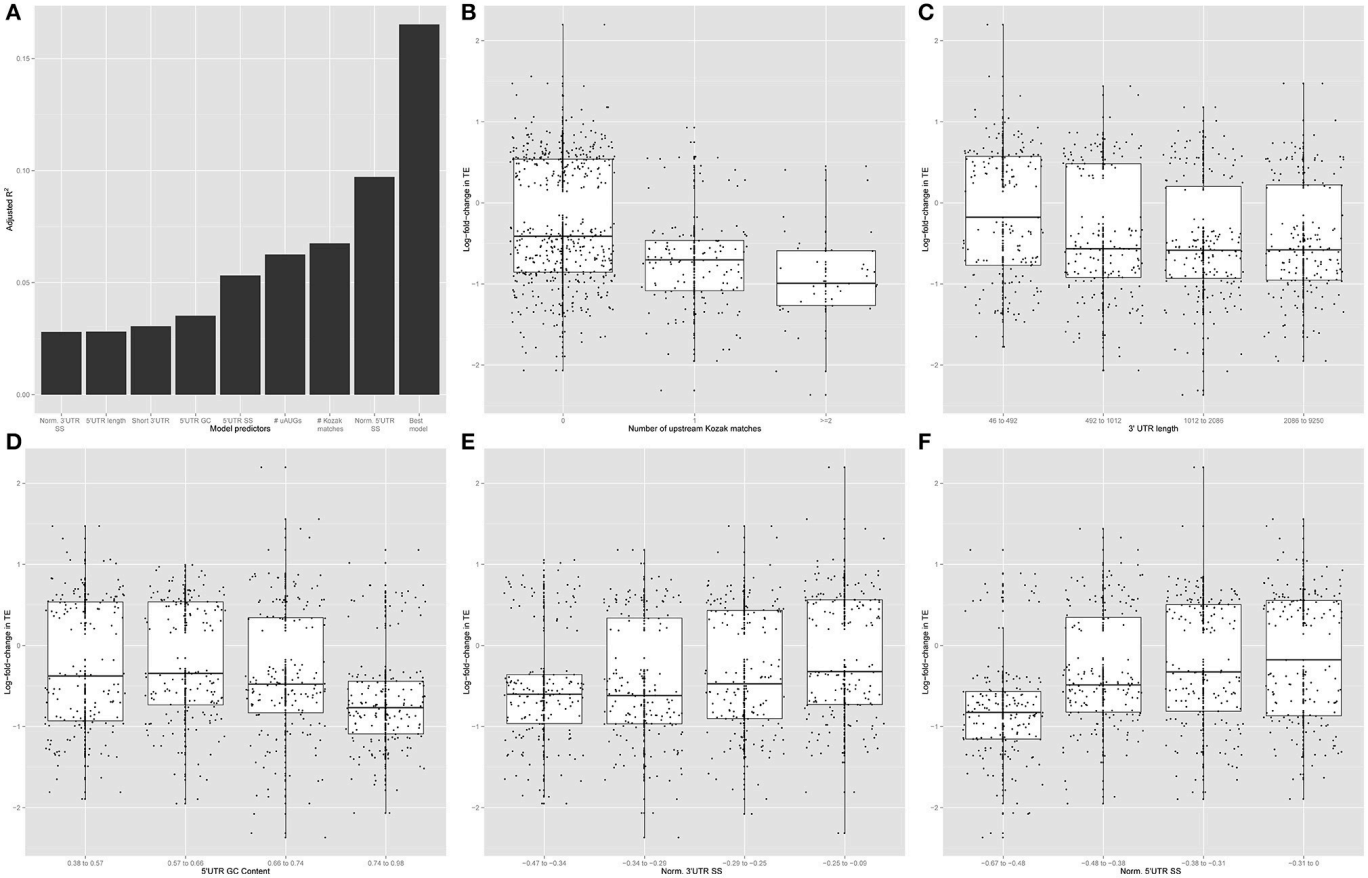
**Primary sequence features alter the response to KCl stimulation. (A)** Normalized 3′UTR secondary structure, 5′UTR length, short 3′UTR, 5′UTR GC content, 5′UTR secondary structure, the number of uAUGs, the number of upstream Kozak matches, and normalized 5′UTR secondary structure score were each significant individual predictor in a univariate linear model (*p* < 0.001). The number of upstream Kozak matches, normalized 5′UTR secondary structure, and short 3′UTR all contributed significantly to the ability of a multivariate linear model to predict the change in CDS TE in response to KCl stimulation. **(B)** Among transcripts that change CDS TE in response to KCl stimulation, more down-regulation of TE occurs with increasing number of upstream Kozak matches (ANOVA *F*-test, *p* = 7.379e-14, *F* = 31.715). Specifically, transcripts containing increasing numbers of upstream Kozak matches are disproportionately downregulated by KCl stimulation (Chi-square test, *p* = 2.6e-11, χ^2^ = 48.713). **(C,D)** More down-regulation of TE occurs with increasing 3′UTR length (ANOVA *F*-test, *p* = 7.069e-05, *F* = 7.3975) **(C)** and 5′UTR GC content (ANOVA *F*-test, *p* = 6.72e-09, *F* = 14.084) **(D)**. **(E,F)** More up-regulation of TE occurs with decreasing absolute value of length-normalized 3′UTR secondary structure (ANOVA *F*-test, *p* = 6.221e-06, *F* = 9.1461) **(E)** and 5′UTR (ANOVA *F*-test, *p* = 3.695e-16, *F* = 26.492) **(F)**.

5′UTR sequence features have been shown to allow for dynamic regulation of CDS translation in response to other stimuli (Watatani et al., 2008; Lohse et al., 2011; Gerashchenko et al., 2012; Young and Wek, 2016). Therefore, we focused on the 642 transcripts showing significant regulation of translation (regardless of changes in transcript abundance) to identify sequence specific mechanisms of this regulation in the UTRs. We tested the hypothesis that primary sequence features of the transcript mediate regulation of CDS TE by KCl stimulation (Figure 5A). Specifically, we tested the predictive ability of linear models incorporating the GC content, length and secondary structure of 5′UTR, CDS, and 3′UTR and the number of uORFs (estimated from either number of uAUGs or number of upstream canonical Kozak sequences). Examining each factor individually, the number of uAUGs, number of upstream Kozak sequences, 5′UTR secondary structure, 5′UTR GC content, 5′UTR length, short 3′UTR (defined as <492 nt), and 5′UTR, and 3′UTR secondary structure normalized by length were each significant predictors (*p* < 0.001) explaining measurable fractions of the change in TE. However, several of these independent predictors were correlated. Thus, in the context of a multivariate linear model, most of the explanatory power only requires three variables: number of upstream Kozak sequences, a short 3′UTR, and 5′UTR secondary structure normalized by length. Overall, the three-variable model predicted ~16.5% of the change in TE, indicating a moderately sized but significant (*p* < 0.001) effect of simple primary sequence features on translation regulation. Model coefficients and statistics are included in Table 1.

**Table 1:** **Linear modeling results**.

**Predictor(s)**	***R*^2^ (adjusted)**	***P*-value**
Number of upstream Kozak matches + normalized 5′UTR SS + short 3′UTR	0.165275	<2.2E-016
Normalized 5′UTR secondary structure	0.097196	3.70E-16
Number of upstream Kozak matches	0.067561	1.36E-11
Number of uAUGs	0.062601	7.67E-11
5′UTR secondary structure	0.053138	2.06E-09
5′UTR GC content	0.035272	9.82E-07
Short 3′UTR	0.030568	4.96E-06
5′UTR length	0.028148	1.14E-05
Normalized 3′UTR secondary structure	0.027991	1.21E-05
3′UTR secondary structure	0.017547	4.48E-04
3′UTR length	0.008997	9.23E-03
CDS secondary structure	0.00768	1.49E-02
CDS length	0.006239	2.53E-02
CDS GC content	−0.00131	6.86E-01
Normalized CDS secondary structure	−0.00155	0.9337

*Adjusted R^2^ and p-values reported for the best multivariate model and for each univariate model*.

Consistent with these results, regulated transcripts were more likely to contain an upstream Kozak sequence than expected by chance, and an ANOVA indicates that TE is downregulated as the number of upstream Kozak sequences increases (Figure 5B). Likewise, we examined the relationship between TE change and the remaining high-performing individual predictors. Binning each predictor into quartiles, we found that more downregulation occurs with increasing 3′UTR length (Figure 5C) and 5′UTR GC content (Figure 5D). Notably with 3′UTR length, the largest change is between transcripts with very short UTRs (<500 bp) and the rest. Additionally, we found that more upregulation occurs with decreasing length-normalized secondary structure in the 3′UTR (Figure 5E) and 5′UTR (Figure 5F). Thus, general features of the primary sequences themselves, particularly the presence of 5′UTR Kozak sequences and secondary structure, can explain a fair proportion translation regulation in response to KCl.

### KCl stimulation alters ribosomal occupancy of 5′UTR

Consistent with our findings above, specific examples of uORFs have been shown, in concert with RNA secondary structure, to serve as regulators of the translation of the CDS (Kozak, 2002; Yaman et al., 2003). Therefore, we examined our RF data to determine whether KCl stimulation also regulated the ribosomal occupancy of the 5′UTR, and whether alterations in ribosome binding in this region was a predictor of changes in translation in the CDS.

**Figure 6:**
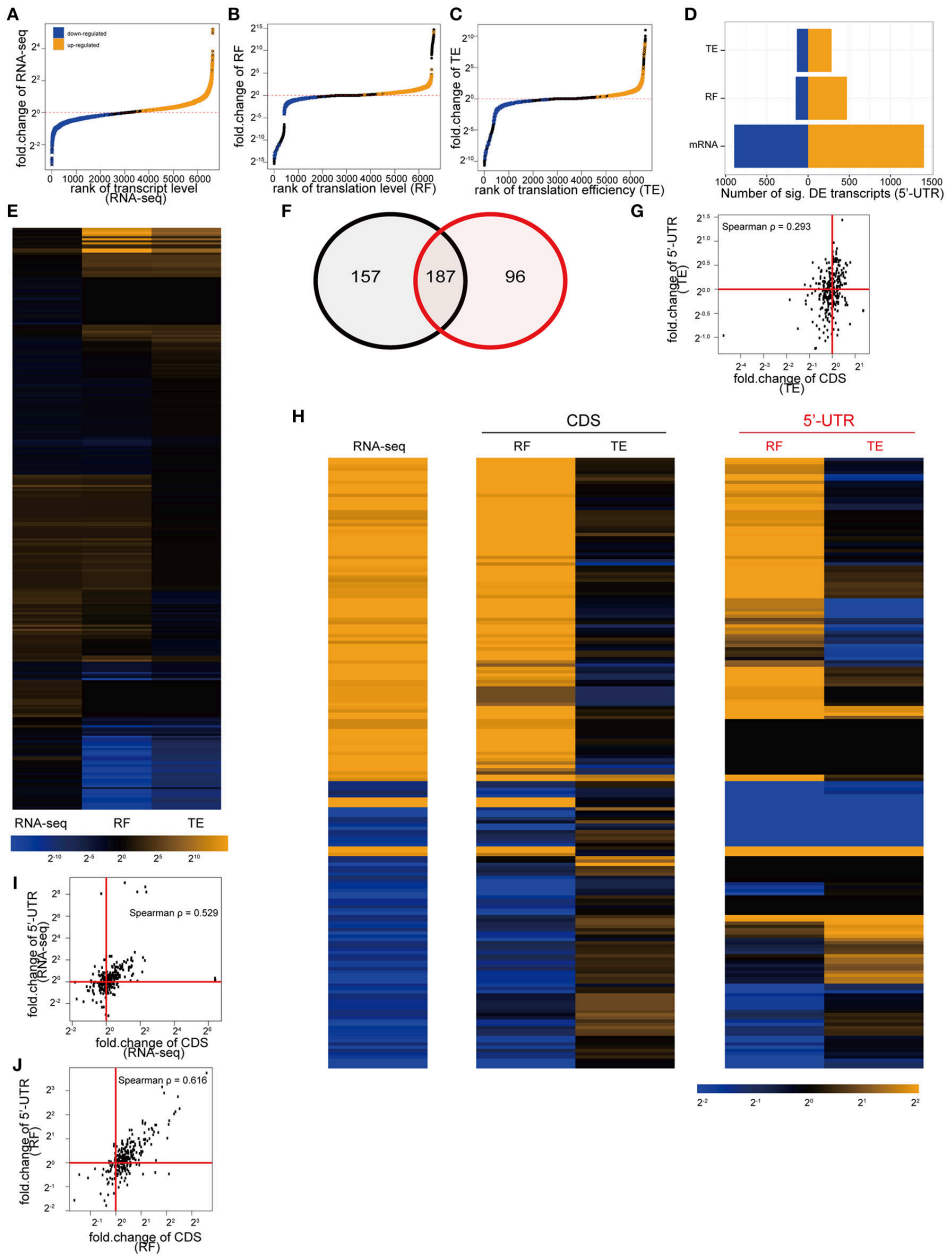
**Depolarization of cultures also alters ribosomal occupancy of the 5′UTR of specific transcripts. (A)** Cumulative distribution showing the alteration of 5′UTR transcript levels by KCl stimulation in culture. One thousand four hundred and five transcripts were significantly upregulated (orange), and 895 downregulated (blue) (all unadjusted *p* < 0.05, permuted t-statistic). **(B)** Cumulative distribution showing alteration of 5′UTR translation levels by KCl stimulation. Four hundred and seventy one transcripts were upregulated, and 149 downregulated. **(C)** Cumulative distribution showing alteration of TE (5′UTR) by KCl stimulation. Two hundred and eighty five transcripts were upregulated, 135 downregulated. **(D)** Bar plot of summary of number of transcripts being up/downregulated. **(E)** Heatmap of log_2_ fold-changes for highly altered transcripts (showing those genes with fold-change in top or bottom 5% for 5′UTR RNAseq, RF or TE, *n* = 283). **(F)** Transcripts showing a significant change in TE in their CDS (black circle) are more likely to also show a significant change in the TE of their 5′UTR (red circle) (*p* < 2.2e-16. Chi-square statistic = 2333, two-sided). **(G)** Scatterplot of TE fold-change in 5′UTR and CDS shows weak correlation. **(H)** Heatmaps for the fold-changes of the 187 overlapping transcripts from **(F)**. There is no consistent relationship in TE fold-change between CDS and 5′UTR across all transcripts. **(I)** Scatterplot of RNAseq fold-change in 5′UTR and CDS shows high correlation. **(J)** Scatterplot of RF fold-change in 5′UTR and CDS shows high correlation.

Similar to the CDS, stimulation resulted in the alteration of ribosomal occupancy and TE of specific 5'UTRs (Figures 6A–D). Furthermore, as with CDS, individual transcripts might be altered at the level of transcription, translation, or both (Figure 6E). We found that a transcript with a significant alteration in TE of the CDS was more likely to also show a significant alteration of TE in the 5′UTR (Figure 6F). Overall there is a weak positive correlation in TE across the two regions (Spearman ρ = 0.29, Figure 6G). However, individual transcripts (Figure 6H) demonstrated different patterns of responses, with some genes showing correlated and others showing uncorrelated or anticorrelated relationships between 5′UTR and CDS TE. This is in contrast to transcript abundance or ribosomal occupancy counts, which are both strongly correlated across the 5′ UTR and CDS (Spearman ρ > 0.52), as expected because of the many transcripts where translational upregulation is passively following an increase in transcript abundance (Figures 6I,J). Thus, while there is a significant overlap between the transcripts showing alterations of 5′ UTR and CDS TE, there is not a simple rule that relates the direction of changes in all transcripts in response to activity.

### FMRP-bound transcripts are disproportionately downregulated by KCl stimulation

Finally, additional regulation may be mediated by the presence of motifs for miRNAs or RBPs. Previously, it has been shown that mir128/128b regulates translation of a variety of transcripts in concert to control neuronal excitability (Lin et al., 2011; Tan et al., 2013). Thus, the concerted changes in translation seen here in response to depolarization might be mediated by a small number of “master regulator” miRNAs targeting sets of transcripts and suppressing their translation. Therefore, we tested whether mir128 or other miRNAs were predicted to disproportionately bind regulated transcripts. In our analysis, several miRNAs were nominally significant, but none of these findings survived multiple-testing correction. While this analysis does not rule out a given microRNA mediating changes in translation for a particular transcript, it does rule out the possibility of one or two “master regulator” miRNAs being responsible for a significant proportion of the changes seen in this study.

**Figure 7:**
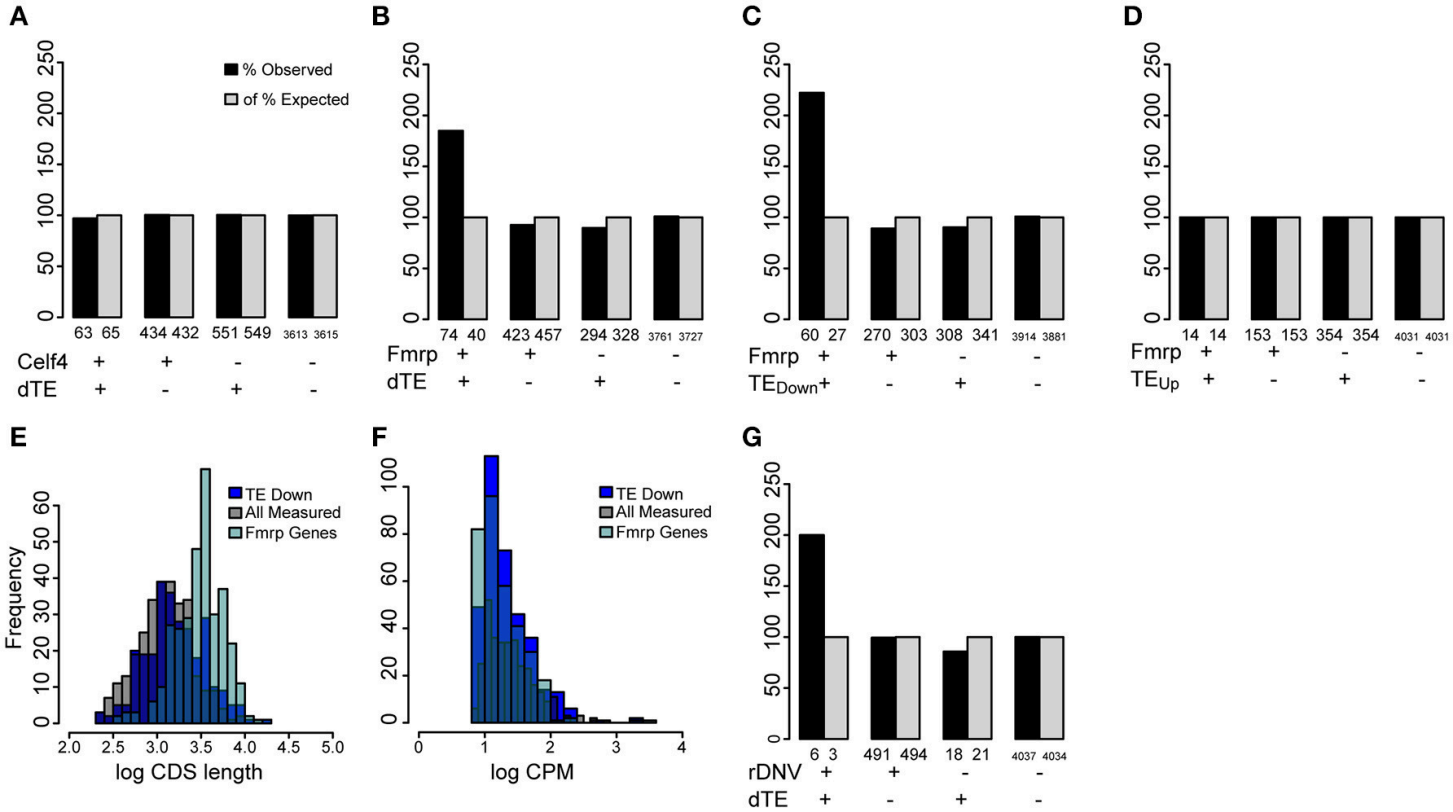
**Regulated transcripts are disproportionately targets of FMRP. (A)** Bar chart illustrating percent of genes overlapping between the translationally regulated genes (significantly changed in TE: dTE+) and published CELF4 targets, compared to expectation by chance. Numbers of observed genes overlapped (*n* = 63), does not differ from chance (Fisher's exact Test, *p* > 0.1). **(B)** FMRP targets significantly overlapped (*p* < 7.7e-08) with genes showing a change in TE (dTE genes, *n* = 71 genes overlapped). **(C)** Specifically, the genes with TE downregulated were found on the FMRP target list ~3-fold more than expected by chance (*p* < 3.2e-10, Odds Ratio 2.97, 95% CI:2.24-Inf), **(D)** while the upregulated genes were not. **(E)** Distribution of CDS lengths (in log_10_) for measured FMRP targets shows they are substantially longer than a random sample of measured genes. The translationally regulated genes (dTE) do not share this bias. **(F)** All three gene categories show similar levels of transcript abundance in the neural cultures. **(G)** Genes associated with autism by analysis of rare *de novo* variation also modestly overlapped with translationally regulated genes (*p* < 0.04).

In contrast to miRNA, binding by RBPs is not easily predicted from primary sequence and must be measured empirically using biochemical approaches such as cross-linking and immunoprecipitation with high throughput sequencing (HITS-CLIP). Notably, two RBPs have been well characterized as regulators of translation in the CNS and analyzed by HITS-CLIP: CELF4 (Wagnon et al., 2012, p. 4) and FMRP (Darnell et al., 2011). Both are widely expressed in the brain and are essential for normal CNS function. Loss of either leads to epileptic syndromes in both mice and humans (Yang et al., 2007; Hagerman and Stafstrom, 2009; Halgren et al., 2012). We tested whether the translationally regulated genes in our analysis are disproportionately targets of these RBPs. While there was no significant overlap with CELF4 targets (Figure 7A), the regulated genes were significantly enriched in targets of FMRP (Figure 7B), and this effect was driven by the downregulated genes (Figures 7C,D). As known FMRP target genes tend to be longer and more highly expressed in the brain than other genes in the genome, spurious overlap with the FMRP targets can occur when candidate genes are similarly biased for length and/or expression levels (Ouwenga and Dougherty, 2015). However, the translationally regulated genes show no such bias (Figures 7E,F). This suggests that these genes are indeed disproportionately downregulated by FMRP. Finally, FMRP targets have been reported to overlap with the 65 recently identified *de novo* deleterious single-nucleotide variants that are associated with autism (Sanders et al., 2015). Therefore, we also checked whether these were significantly enriched amongst the regulated genes. We indeed found a significant 2-fold enrichment, though only 24 of the ASD genes were robustly measured in our cultures, so the number of genes overlapped is modest: these include *Tcf712, Phf2, Wdy3, Dnmt3a*, and *Mib1* (Figure 7G).

## Discussion

In this study, we performed genome wide nucleotide-level analysis of transcription and translation to quantify the extent of translational regulation in response to sustained KCl stimulation. It is well established that neuronal depolarization triggers the transcription of activity-depended genes (Ghosh and Greenberg, 1995; West and Greenberg, 2011) and this was also seen in our study. However, in our study, substantially more genes were regulated post-transcriptionally: we found that nearly 36% of the robustly measured transcripts altered ribosomal occupancy following neuronal activity, and 10% of transcripts significantly changed their TE, thus showing regulation of translation independent of mRNA abundance. Indeed, 40% of the translational variance overall could not be explained by changes in mRNA abundance. These findings suggest that the translational machinery has substantial regulation independent of transcription.

We also found that the change in TE of an mRNA depends partially on the cis-regulatory elements present in the sequence itself. Features such as 5′UTR secondary structure and uORFs each independently accounted for more than 5% of the variance in this regulation. In a combined model, these and other features could account for 15–20% of the change in TE. These features largely acted additively: thus, two or more cis-regulatory elements driving the TE in the same direction may couple so as to achieve a greater control over protein synthesis. Our model did leave a large fraction of the change in TE unexplained. Given the detection of an enrichment of known FMRP targets seen in our analysis, we think the remaining fraction may be attributable to other RBPs (Abaza and Gebauer, 2008) or translation initiation mechanisms that circumvent standard regulatory elements (Komar and Hatzoglou, 2011; Paek et al., 2015). One distinct possibility is that a number of these transcripts share regulation by Eif4e—a known regulator of translation initiation in response to cellular stress. Indeed mutations impacting this regulation have been shown to cause autism like phenotypes in mice (Gkogkas et al., 2013). However, only the targets in fibroblasts have been identified thus far (Mamane et al., 2007), and these do not overlap significantly with the regulated genes detected here (not shown), though baseline differences in transcript expression between fibroblasts and neurons make this an imperfect analysis. Nonetheless, our study provides a resource for further modeling as the regulatory targets of Eif4e and other RBPs in the CNS are identified. We also found that TE was more downregulated in those transcripts with increased 3′UTR length. It is interesting to note that prior work suggested a similar depolarization paradigm led to a shortening of 3′UTRs by selection of an earlier polyadenylation signal (Flavell et al., 2008). If longer UTRs lead to deceased translation during depolarization, subsequent use of an earlier polyadenylation signal could serve as a homeostatic mechanism to allow for the recovery of translation levels.

Our data revealed that the overall translation is reduced in response to neural depolarization. Of the transcripts exhibiting activity-dependent change in TE, 64% showed a reduction in TE. This finding is consistent with a previous study which, using [^35^S]Methionine labeling and polysome profiling, reported 15–30% decrease in global translation after 2 h of neuronal depolarization with 50 mM KCl (Krichevsky and Kosik, 2001). Also in line with our finding is a recent study which used the RF/RNAseq approach and reported a pervasive translational downregulation in the hippocampus after 30 min as well as 4 h after fear learning (Cho et al., 2015). Translation of proteins, generation and propagation of action potential, and reversal from the depolarization-induced high cytoplasmic [*Ca*^2+^] are all extremely energy-demanding processes (Buttgereit and Brand, 1995; Attwell and Laughlin, 2001; Clapham, 2007). Therefore, the reduction of translation during neuronal activity may be a homeostatic response to allow devotion or more resources to repolarization. Interestingly, translation downregulation is also a hallmark of seizure, a disease characterized by sustained neuronal excitation (Fando et al., 1979; Collins and Nandi, 1982). Future studies monitoring activity-dependent translation across different time points for a prolonged period may reveal differences between physiological and pathological translational dynamics.

One important feature of our study is the inclusion of glia in neuronal culture. We believe that this allowed glial modulation of neuronal activity, as is the case *in vivo*, thus making our findings more physiological. On the other hand, it imposed a major limitation on our study by causing many neuronal genes to be excluded from analysis because of low read counts. In part, lack of sensitivity for low abundance transcript has as much to do with the inherent challenges of RF library preparation as it does with the presence of glia. In RF experiments more than 80% of reads are frequently consumed by rRNAs (Ingolia et al., 2009, 2012). Regardless, having multiple cell types present challenges to interpretation as the upregulation of a transcript by RF or RNAseq in one cell type could be nullified by a stronger downregulation in another. We presented some findings computationally separating transcripts to those likely derived from either neurons or glia based on their specificity in *in vivo* profiles, and discovered evidence for a greater degree of translational regulation in neurons. However, true confirmation of this finding awaits future studies leveraging either more purified cultures or cell-type specific assessments of translation *in vivo*. In sum, our current findings describe the holistic effect of KCl on neuron/glia mix culture.

Finally, our data also provide a resource to considering the response of individual transcripts to a strong depolarizing stimulus. Across the genome, we detected transcripts showing numerous combinations of mRNA level, ribosomal occupancy and TE changes following neuronal depolarization. These are provided as a supplemental Table (Table S2).

## Author contributions

JDD and JSD conceived the project and designed the study. JSD performed RNA-seq and Ribosome Footprint experiments. CY, AL, JDD, and DO performed computational and statistical analyses. DS provided experimental support. DS, AL, JSD, CY, and JDD interpreted results and wrote the manuscript.

### Conflict of interest statement

The authors declare that the research was conducted in the absence of any commercial or financial relationships that could be construed as a potential conflict of interest.

## Supplementary material

Figure S1 **Transcript abundance is less predictive of ribosome occupancy for neuronal transcripts than for glial transcripts**. Transcripts were subsetted into neuronal and glial specific lists as described in the methods. **(A,C)** mRNA abundance predicts only 43% variance in ribosome occupancy at baseline and 53% variance in translation following KCl stimulation in neurons. **(B,D)** mRNA abundance predicts more than 65% of the variance in ribosome occupancy at baseline as well as following KCl depolarization in glia.

Table S1 **Transcript ID, Gene symbol, and likely originating cell type for each transcript, based on pSI calculation from Zhang et al. (**2014**)**.

Table S2 **RNA-seq, RF, and TE levels without (noKCl) and with (yesKCl) depolarization**.
