# Supplementary figures and images for: Quantitative Nucleotide Level Analysis of Regulation of Translation in Response to Depolarization of Cultured Neural Cells
Source: Front Mol Neurosci. 2017 Jan 27;10:9. doi: 10.3389/fnmol.2017.00009 (PMC5269599; doi:10.3389/fnmol.2017.00009)

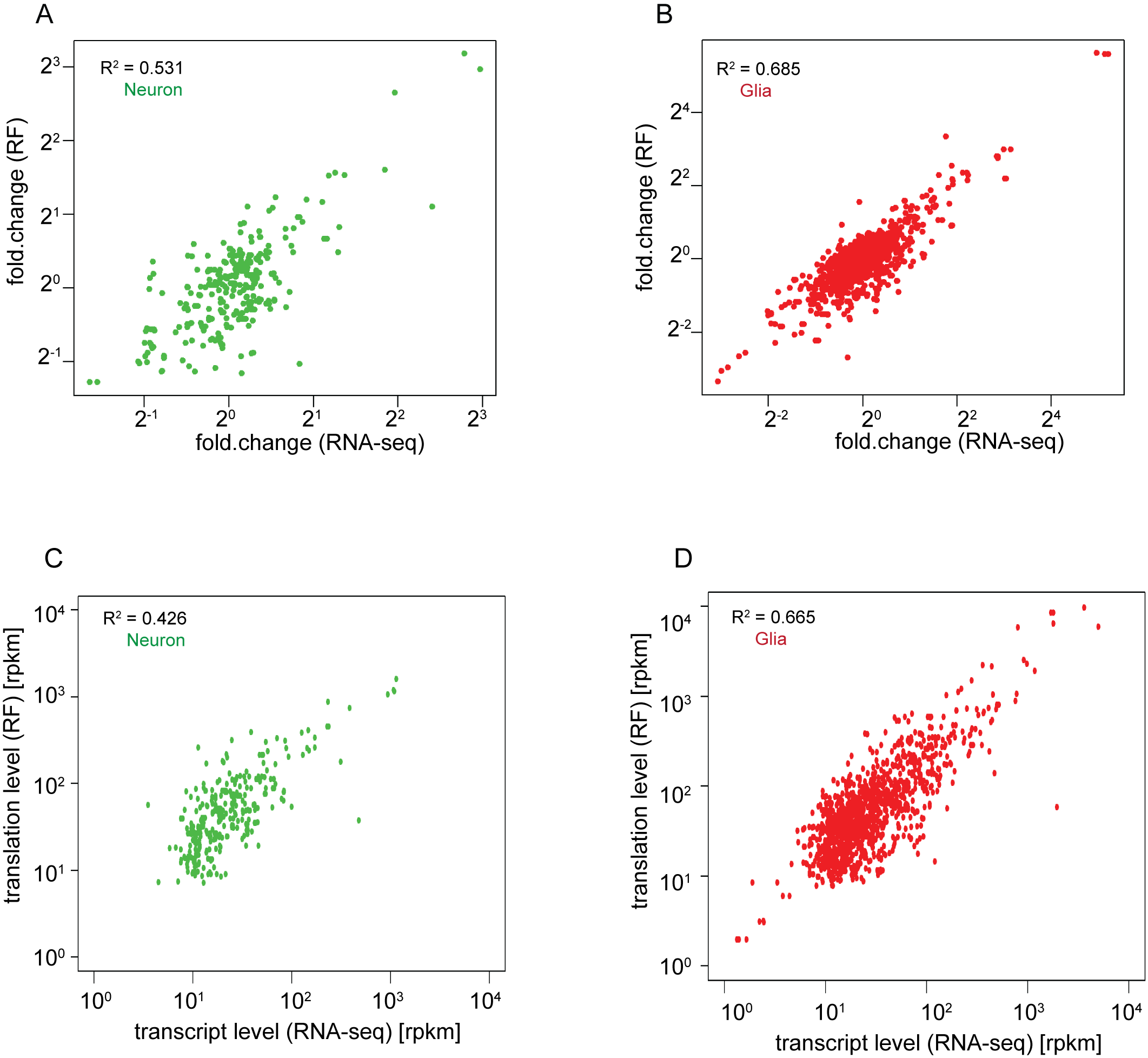

Supplement: Figure S1 — Transcript abundance is less predictive of ribosome occupancy for neuronal transcripts than for glial transcripts. Transcripts were subsetted into neuronal and glial specific lists as described in the methods. (A,C) mRNA abundance predicts only 43% variance in ribosome occupancy at baseline and 53% variance in translation following KCl stimulation in neurons. (B,D) mRNA abundance predicts more than 65% of the variance in ribosome occupancy at baseline as well as following KCl depolarization in glia. [file Image1.TIF]
